# Interventional treatment of basilar trunk artery aneurysms associated with situs inversus totalis: A case report

**DOI:** 10.3389/fsurg.2022.971340

**Published:** 2022-08-30

**Authors:** Tianlin Long, Quanhua Xu, Xu Chen, Yan Ma, Yuanjian He, Jia Rao

**Affiliations:** ^1^ Department of Neurosurgery, Bijie Traditional Chinese Medicine Hospital, Bijie, China; ^2^ Department of Neurosurgery, Weining County People’s Hospital, Weining, China

**Keywords:** basilar trunk artery aneurysms, situs inversus totalis, interventional treatment, angiography, clinical cases

## Abstract

Basilar trunk artery aneurysm (BTAA) has an overall low incidence in intracranial aneurysm, but its rupture is associated with high morbidity and mortality in older people. Situs inversus totalis (SIT) is a rare congenital abnormality characterized by visceral rotation and vascular abnormalities. It has been described in several uncommonly clinical cases, along with middle cerebral artery aneurysms and large carotid cavernous aneurysms. However, the association between interventional embolization for BTAA and SIT has not been reported. We described the angiography findings and interventional treatment of the association of BTAA with SIT.

## Introduction

With a 2.1% incidence in intracranial aneurysms, basilar trunk artery aneurysm (BTAA) is a rare lesion (1, 2). The four subtypes of BTAA (acute dissecting aneurysm, segmental fusiform ectasia, saccular aneurysm, and chronic mural bleeding ectasia) can be distinguished based on imaging symptoms and pathological characteristics (1, 3). BTAA is mostly recognized in male patients above the age of 60 years (1, 2). The following types of BTAA are common aneurysms (2, 4): (1) asymptomatic, usually accidental, or physical examination; (2) sudden headache, nausea, vomiting, and other subarachnoid hemorrhage symptoms; and (3) focal neurological impairments related to aneurysm compression or effects on the brain stem. Endovascular therapy and aneurysm clipping under a surgical microscope are now used to treat BTAA (4).

Situs inversus totalis (SIT), a rare congenital anomaly with a 1:10,000 incidence and a frequency of 1.5:1 in males, is defined by a mirror-image transposition of both the abdominal and thoracic organs (5, 6). It has been characterized by a number of unusual clinical cases and aneurysms (large carotid cavernous aneurysm and middle cerebral artery aneurysm) (7, 8). Interventional embolization for BTAA and SIT, however, has not been linked in any studies. In this report, the uncommon relationships and technical challenges of endovascular therapy are described.

## Case description

**Figure 1:**
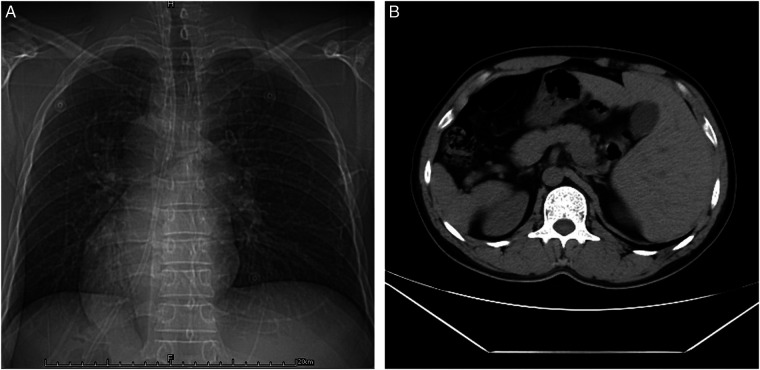
CT confirmed the patient with SIT. (**A**) Chest CT presented a right-sided view of the heart. (**B**) Abdominal CT suggested left–right viscera inversion.

On 12 May 2020, a 42-year-old man with no notable medical history was admitted to the hospital for “sudden onset of headache for 16 h and aggravation with coma for 5 h.” The results of the physical examination were as follows: coma, bilateral round pupil size, diameter of about 2.0 mm, light reflection gone, cervical rigidity, percussion and auscultation with dextrocardia, and a temperature of 36.8°C. He underwent head computed tomography (CT) for Hunt–Hess III and subarachnoid hemorrhage. Figure 1 illustrates how CT further established the patient's SIT. Figure 1A shows a right-sided image of the heart on chest CT, and Figure 1B shows a left–right visceral inversion (Figure 1B).

**Figure 2:**
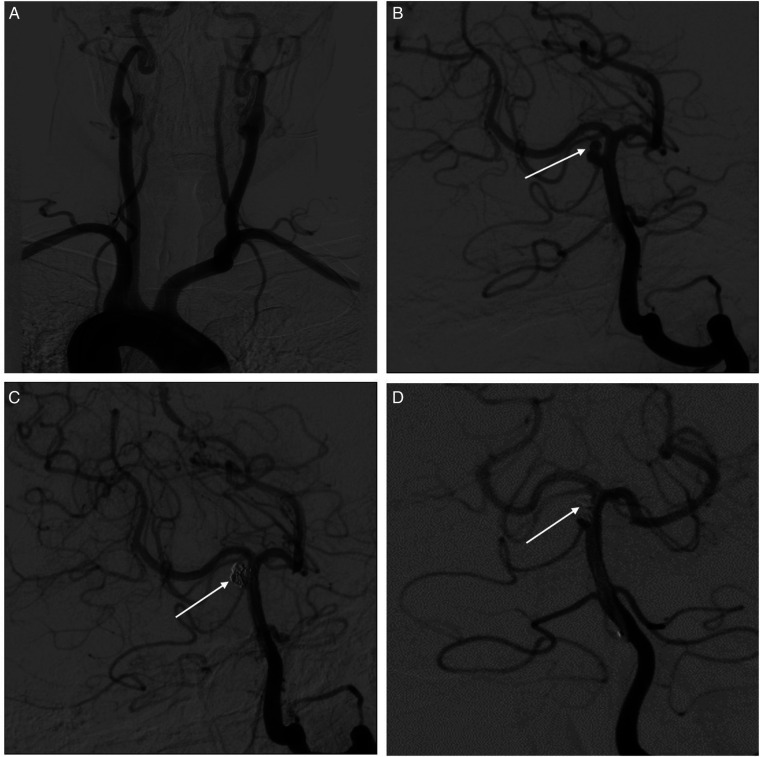
Interventional surgery was performed to treat BTAA. (**A**) Anteroposterior and lateral angiography of bilateral common carotid arteries and bilateral vertebral arteries shows the inverted arrangement of the aortic arch and its upper vessels. (**B**) An aneurysm (white arrows) in the middle part of the basilar artery, with a diameter of about 3 mm  ×  4 mm, and the apex daughter tumor. (**C**) Arrow indicated successful aneurysm embolization after interventional therapy. (**D**) Re-examination of cerebral angiography showed a small part of tumor neck residue (arrow indicated) one year after the operation.

The day after being admitted to the hospital, the patient had emergency cerebral angiography and had an intracranial aneurysm embolized. A modified Seldinger puncture was made on the right femoral artery, and a 6F arterial sheath was introduced while the patient was lying in the supine position. The 150-cm superloach guide wire and a 5F single-bend angiography tube were used to perform anteroposterior and lateral angiography in the bilateral common carotid arteries and bilateral vertebral arteries. The abdominal aorta, ascending aorta, and aortic arch were all pointed in opposing directions, according to the data. The left common carotid artery and the left subclavian artery were identified from an unidentified trunk that was situated on the side of the ascending aorta (Figure 2A).

An aneurysm was detected in the middle part of the basilar artery, with a diameter of about 3 mm × 4 mm, and an apex daughter tumor was formed (Figure 2B). The 150-cm superloach guide wire with a 6F guiding catheter was inserted into the middle part of the left basilar artery, followed by high-pressure heparin saline for continuous and stable perfusion. The aneurysm was confirmed by 3D imaging to find the working angle. The Avigo-14 microguide wire with an Echelon-10 microcatheter was inserted into the aneurysm cavity under the direction of the road diagram. The microguide wire was withdrawn, and the first spring coil could not form a basket because the tumor neck was wide, so the Avigo-14 microguide wire with a Rebar-18 stent catheter was inserted into the basilar artery apex. Then, a Solitaire-AB (4 mm × 15 mm) stent was released smoothly through the stent catheter, which covered the tumor neck satisfactorily and was sent into the spring coil sequentially through the microcatheter until the embolization degree was satisfied (Figure 2C). Finally, anteroposterior–lateral angiography showed that the aneurysm was completely embolized, the stent was successfully released, so the operation was completed, and all catheter system levels were removed. The patient recovered and was discharged after routine postoperative treatment, and his physical condition was generally good. Re-examination of cerebral angiography showed a small part of tumor neck residue 1 year after the operation (Figure 2D).

## Discussion

SIT develops a disorder of visceral rotation during embryonic development, which is associated with mutations at a site in the parents' genes. The viscera are in opposite directions but do not affect their function and ordinary life. Few cases of SIT have been reported with surgical experience at home and abroad. The case of cerebrovascular interventional surgery in SIT patients has not been reported, but there are reported cases of cardiovascular interventional surgery and open surgery (9, 10). Matsuno et al. reported the association between cerebral aneurysms and the transposition of the aorta, and the surgical clipping of the aneurysm was carried out *via* the right frontotemporal craniotomy on the next day after admission (7). However, when the SIT patients require surgical treatment, the antiphase organ structure will increase the difficulty of diagnosis and surgical treatment (6). The rare BTAA has complex etiology, and its rupture will cause subarachnoid hemorrhage, which will seriously affect the patient's life safety and prognosis. At present, endovascular intervention or craniotomy is commonly used in the treatment of basilar aneurysms. A craniotomy is generally performed by clamping and vascular bypass (11). Interventional surgery usually uses aneurysm embolization and vascular remodeling devices. Wang et al. reported that 11 cases of basal aneurysms were treated with endovascular intervention (eight cases were unruptured and three cases were ruptured), and no serious complications occurred during or after surgery (12). This case was considered to be the result of flow impingement at the bifurcation of the basilar artery and the long circumflex branch of the brainstem. Considering the complex structure of BTAA and the difficulty of operation, interventional therapy was used in this BTAA associated with SIT patients, and we summarized it as follows: (1) The majority of patients with left 30° before projection imaging can better show the aortic arch in form, but this case the reverse right front 30° oblique imaging. (2) The judgment and thinking operation are on the opposite point of view: because the abdominal aorta is located in the right, the operation began to consider that is from the opposite direction from below, and the ascending aorta and aortic arch are opposite direction structures. The left subclavian artery and the left common carotid artery arise from the innominate trunk on the side of the ascending aorta, and the aortic arch structure on the contrary, but the performer was still in operation. Then, the doctor should adjust the operation at any time to overcome many adverse factors such as reverse catheterization, reverse operation, and inverse radiographic image. (3) For cases of abnormal anatomical structure, the familiar and easy operation should be used as far as possible; under the guidance of reverse thinking, the brain, eye, and hand are highly coordinated to maximize the safety of patients during the operation. (4) In addition, the long-term efficacy of interventional therapy for SIT with BTAA remains to be followed up.

## Conclusion

BTAA associated with SIT being successfully treated with an interventional treatment is reported for the first time in the literature. The complex aortic arches in these SIT patients should require special attention during endovascular surgery.

## Data Availability

The original contributions presented in the study are included in the article/Supplementary Material, further inquiries can be directed to the corresponding author.
